# Mobility and interlinkage: the transformation and new approaches for anthropological research

**DOI:** 10.1186/s41257-022-00072-x

**Published:** 2022-08-29

**Authors:** Daming Zhou

**Affiliations:** Centre for Migration and Ethnic Studies, Sun Yat-sen University, Block 410, Southwest District, Southern Campus, No.135, Xingang West Road, Haizhu District, Guangzhou, China

**Keywords:** Mobility, Interlinkage, Anthropology, New approaches, road research, internet anthropology

## Abstract

Mobility and interlinkage have become the most important characteristics of our time. The mobility and interlinkage of people, material and information constitute the way and rules of the operation of today’s world. Internet links, cloud computing, complex database and human computation have changed the way people relate to the world, thus the anthropology for understanding and interpretation of human cultures have changed correspondingly. Cultures in the state of mobility and interlinkage, such as spatial changes, the evolution of interpersonal relationships and the new cultural order, have become a new subject.

## Introduction

Sun Yat-sen University established the National Supercomputing Guangzhou Centre about 10 years ago, and developed the Tianhe-2 supercomputer system. Tianhe-2 has set many world records in computing speed. From 2013 to 2015, Tianhe-2 was among the top 500 supercomputers. It ranked first in the world for six consecutive times on the ranking list, becoming the first supercomputer in the world’s supercomputing competition to win six consecutive championships, breaking the world record in the field of supercomputing. Why is it the fastest computer in the history of supercomputing? Because Tianhe-2 has approximately 17,920 computing nodes, each with two Xeon E5 series 12-core CPUs and three Xeon Phi 57-core coprocessors (computing accelerator cards), with a total of 3.12 million computing cores (Zhang 2013). This way of linking determines its computing speed.

Through the above phenomenon, many puzzles that have confused the author before have been solved at the same time, because I realize the importance of mobility and interlinkage in the information age, and the two concepts are closely related. As we all know, the earliest method of computer connection may be the electronic method, now it is the optical method, and the next step is the quantum method. A lot of people are talking about quantum computers - is the quantum link almost instantaneous? Will it be faster than the speed of light? These are all about the connection method. I am not trying to explain the technology here, but to see other things through such a phenomenon.

Associated with the forementioned phenomenon, as of our physical body, Traditional Chinese medicine (TCM) emphasizes the meridians, each acupoint is the connection point of a certain meridian. As long as any acupuncture point is blocked, the entire human body will get sick or even die. This is what life is like. Looking at the mobility of people from a social perspective, high-speed railways, airports, highways and other infrastructure are being built on a large scale. Mobility is speeding up under these circumstances. How is mobility linked? What are the characteristics, social and cultural consequences? These are issues that anthropology, history, and other humanities should pay attention to.

How is the mobility of people linked? In the past, the academia did not pay attention to this issue. The author noticed this matter through various perceptual empirical phenomena, as a subjective argument. I just hope to raise this question and provide a path to rethink, which can be approached by various disciplines. I believe the content discussed in this article is also an inconclusive academic exploration to a certain extent. I believe it will also inspire us to think about related issues, such as why we should emphasize the continuity of Chinese civilization. Chinese historians have proudly emphasized that the Chinese civilization has continued in the past five thousand years and has not broken. So how are the historical nodes of each dynasty and region of Chinese civilization linked? This is an important topic for historians. Nowadays we are studying civilizations. Prof. Kwang-chih Chang categorized the civilization of the whole world into two types: One is the discontinuous civilization, and the other one is the continuous civilization (Chang 1986). Chinese civilization and Mayan civilization emphasize the unity of nature and man. European civilization is a discontinuous civilization, which is not a normal state. Continuous civilization is a normal state. The fact that Prof. Kwang-chih Chang can be recognized by the academic community has a lot to do with him putting forward a view which is different from that of the Western scholars. In fact, what he said is a way of linking within civilization.

## Research method

Although anthropology did not have special research on interlinkage in the past, it is still possible to find similar discussions, such as “cultural contact”, “cross-cultural communication”, “ethnic interaction”; “social networking” studies explore interpersonal relationships, which is exactly how people link and form networks. The “road research” that has emerged in recent years is to study the mobility and interaction of people through the connection between roads and settlements. Another is Internet Anthropology, which focuses on how to deal with the changes brought by the Internet to interpersonal relations and the relationship between people and the outside world.

Changes in transportation methods, including changes in roads and means of transportation, and the rise of the information superhighway (Internet), have affected the mobility of people, materials, and information in our contemporary society. The anthropological research methods that keep pace with the times are “road research” and “Internet anthropology”. The first is about the roadology. There are the research paths: First, the road is used as an infrastructure. Although the road is artificial, once it is formed, it becomes a part of the natural ecological system. Human society links with road to settlements. The way of connecting roads has a profound impact on the prosperity of settlements and people’s lives; the second is to see roads as a special space, and study it by combining mobility with sociality, roads and vehicles, traffic rules, behaviours, and living styles. On the one hand, it can break the physical, geographical and political barriers and expand the space for social interaction; on the other hand, it can accelerate the interaction between different spaces and shorten the time required for social interaction. Many things are contradictory. The establishment of express transportation is to shorten the transportation time, but because of the convenient transportation, the arrangement of various things is more frequent, which makes people more exhausted. The third is the road study as a kind of regional research, which is an approach proposed by myself. I regard the study of roads as a kind of regional research, and look at a region from a large transportation network. Why should there be a regional vision? I regard the “river basin” or “spring domain” as a region, and the “road domain” can also be regarded as a region. In a “road region”, the settlement located at the hub of transportation often becomes the regional centre, and the regional centre is connected with other regional centres, and at the same time radiates to the settlements at all levels in the region through roads (Zhou 2019a).

Another is Internet anthropology research approach. First of all, the traditional anthropological research approach in the Internet age seems impossible to conduct. First, it is difficult to determine the boundaries of a field space. The community we studied in anthropology in the past had boundaries, but the space of the Internet seems to be a borderless and open structure. The second is the change in the relationship between researchers and investigators, from face-to-face to a virtual relationship. The third is the change in the present ethnography. Different from the previous fields, the network is an open structure, which is extremely extensible and dynamic at the same time. What is more important in network research is that not only many nodes can be seen from the network, but also the relationship between nodes can be clearly revealed, so network research can do better in presenting movements and changes. Network ethnography discovers the interactions and differences between cultures through the study of nodes and their relationships. The fourth is the change in participation in observation. There is a big difference between observations in nature and observations on Internet. The new instruments such as “electronic eyes” can be used for observation. Fifth, the authenticity of Internet data is hard to check, as far as the age, gender, and nationality of the person, all difficult to make authenticity judgments, especially since we are now in a virtual community. The sixth is the problem of context in Internet ethnography. The context that has been emphasized in the past paying attention to the surrounding environment and atmosphere of the research object. However, Internet ethnography may only analyse the discourse and text itself, and there is no way to analyse its context (Zhou 2019b).

## Results and discussion on mobility and interlinkage

Mobility, a classic theme of anthropological study.

Anthropology has been concerned about human mobility since its birth, and migration is an important research theme.

Mobile phones have become an inseparable part of modern people, especially when we travel during the covid-19 pandemic. For example, no one can travel easily in China without a health code, which generated on each person’s mobile phone. The airport requires the presenting of the health code to enter as well as to leave. The Internet brought us convenience, but it also brings us great trouble. The younger generations use computers and mobile phones much better than our generation (born in the late1950s) or older people. Our generation has not been influenced by this set of electronic products, has not installed many applications, which brings great inconvenience for senior people also. Now when we travel, many services are automated. We have suddenly entered the electronic era, but some senior people, even with the possession of the electronic devices, cannot catch up with the tide. The confusion caused by technologies is a daily scene in major cities, even more common in small town, but less attention was drawn to.

Let’s return to anthropological research on mobility. With the mobility of people, materials, capital and culture are all moving. Anthropological researchers can use technology to observe the frequency, direction and scale of population migration, from which we can see where the economy is active. This is also the dialectical relationship between population mobility and urban vitality. We should take population migration as an important indicator to measure the vitality of a city.

The mobility brought about by the Internet has transformed China into a digital country.

For instance, digital culture, digital government affairs, digital living and digital industry are all concrete manifestations of digital China. Behind all digitalization is the question of how to store, manage, process and link. After the technological evolution, the academic community has not put forward the corresponding theoretical framework and analysis concept. The Japanese made the concept of “blockchain”. We connect the single “block” of the multivariate data layer with a chain. It has been a decade since the emergence of blockchain, and now it has become the most important concept and even infrastructure for the foundation of the entire Internet platform. Sometimes a new concept, a new path, is so important that it leads the world trend of related research.

How do we externalize the understanding of interlinkage? For example, as in a library, how do we pick up the book among millions of collections. We have a set of book classification system, and at the same time use a computer to develop a programming system, so that readers can input the keywords or the first letter of the bibliography into the computer, or input the author’s name, to find the book one need. Of course, this depends on two factors: one is that the search system itself is large enough to include the books you want to search for, and the other is that you can master this set of search methods. Today, in this era when everyone is the medium, every platform and even everyone may produce massive amounts of data. When it comes to huge data processing, the first and most important thing is to save it, and secondly, it may also involve confidentiality, privacy and security. That is to say, in today’s Internet ecosystem, the openness and security of data is an extremely important issue, and blockchain is one of the best technologies that can well solve distributed trust and information security that cannot be tampered with. China’s digital infrastructure construction is now facing a huge problem, that is, we have entered the 5G era, there are very good technologies that can make everyone’s information processing capabilities more efficient, just like everyone is a sports car with super performance, but the current distribution of China’s digital infrastructure is uneven, some are highways, some are mud roads, running on a muddy road, no matter how good a sports car is, the road is rotten and the links are not smooth, the overall speed is low. Therefore, the importance of interlinkage method revealed.

Interlinkage: disappearing boundary between physical and digital world.

In today’s China, interlinkage is making our lives connected closer than ever, we have more convenient means of transportation, and we have more intelligent digital management methods. Of course, another important thing is that the Internet links all products and individuals. In the past, what we saw was to simply link between people and people through technology, but today we see that the technology represented by the Internet of Things has made a more comprehensive and active link between people and matters. This is more complicated, and it reflects not just the dissemination and reception of information, but also the use of links to create more scenarios to serve the people. Another is the integration of the whole value chain. We not only want to spread, but also integrate production, management and service, which is the goal of today’s industrial Internet. Now we are working on smart cities, and several major Internet companies in China have entered the market one after another to do technical research and development and operation on the infrastructure of smart cities. The link between people and the city has begun to extend from the real physical space to the digital space. For example, a household refrigerator can accurately and individually recommend the food calories and related recipes you need for each meal based on your consumption habits and behaviours, as well as matched health big data; manufacturers of new energy car can track the location of your car based on the sensors on the car, and transmit data on the running status of the car in real time according to the sensors to ensure safer driving.

This is an opportunity and a challenge for every discipline as the physical and digital worlds are increasingly integrated. For example, more and more academic articles are published every year, and a large part of the reason is that because of the convenience of searching data, the tools for processing data have become more efficient. But it is also a challenge. Now we are not worried about finding data, but how to deal with massive data, which requires mastering new methods of processing data.

How can we make a full range of integration? There are three driving forces: Linking, Computation, and Interaction. For example, in the simplest case, you order breakfast, which involves cooperation with many institutions, personnel and scenarios. Although this is a small daily behaviour, if you think about it from the perspective of technical implementation, it involves a lot of interaction, calculation, and the core of these calculations is to realize the link, which is actually the digitalization process of the simplest human behaviour logic. Human thinking is linked, and we also need to have coherence. For example, if you cannot write an article, it is because your minds are not well connected.

Now you can see that the link is everything and is the basic guarantee of fast flow. To achieve a fast mobility, you must first have a stable and fast connection, we have invisible links — the Internet, we also have visible links — fast traffic. Fast transportation links break the traditional market system, spatial network relationship, and power and political structure. For example, on Kuaishou, a live-video streaming APP, the live-video team of “Seven Fairies of the Dongs”, which is composed of a group of girls from the Dong ethnic group, uses short videos on the Internet to promote ethnic culture and help their hometown get rid of poverty. This is a new way of connecting, which was simply unimaginable before Internet technology.

Back to an anthropological perspective. With the emergence of new things in the new era, there will be many new topics, such as new links and changes in urban and rural structure. Our early cities were built along rivers without exception. For example, Chongqing, because the Yangtze River and Jialing River converged in this place. This used to be a very important wharf. Along the Yangtze River, wharfs connected cities one by one, such as Hankou, Jiujiang, Nanjing, all the way to the downstream Shanghai, leaving a large number of towns on both sides of the river, which is the spatial structure presented in old linking methods. But with the advent of roads and railways, especially at the intersection of railway jointing, important towns are formed. An example that the author often cites is Zhengzhou and Kaifeng. Kaifeng was both the centre of China and the enter of Henan Province in the era of river transport, which “Riverside Scene at Qingming Festival”, the famous ancient Chinese painting, depicts as very prosperous. In the past, Zhengzhou was not a provincial capital, so Henan University was not in Zhengzhou, but in Kaifeng. Later, due to the construction of the Beijing-Hankou Railway, which passing through Zhengzhou, Zhengzhou became the central city of Henan Province. Finally, a series of cities formed along the railway line. Another example is Changsha, Zhuzhou, Xiangtan in Hunan, because Zhuzhou became a railway junction, and finally became a prefecture-level city from a town, and surpassed Xiangtan. Changes in transportation methods have changed the centre of the city and the centre of settlements. These settlements are actually links one by one, and the change of the location of a town will inevitably change its urban-rural structure and network.

Now with the advent of the high-speed rail era and the aviation era, many things are unpredictable. How will our towns be laid out and how will they develop? The author believes that the arrival of high-speed railways and highways, and even the aviation age, will make cities larger and larger, and megacities more and more “mega”. New links, changes in the urban-rural structure, and changes in cities and towns will lead to changes in the mobility of people, goods, and capital. For example, Chongqing, which has been a municipality directly under the Central Government for a short period of time, but it is in this short time that Chongqing has developed rapidly. It is said that there are currently 20,000 high-rise buildings in Chongqing, ranking second in China. Why? Because Chongqing is a transportation hub, a shipping hub, a hub for high-speed rail, and a hub for aviation, it is developing very fast. Good educational and medical resources have begun to concentrate, and there is a trend of further concentration.

Correspondingly, people’s perception of urban and rural areas in the digital age will also undergo great changes. With the help of digital technology, things that could only be felt in cities in the past can now also be felt in the countryside, which is almost synchronous. We can see villagers in the countryside of Chongqing talking about the debate about the US presidential election. This is the universal benefit and dividend brought by the popularization of technology. Therefore, from this perspective, many people have inaccurate acknowledgement that Kuaishou is a platform for the country people. Nowadays, can we still use the dualistic thinking of the past to distinguish between urban and rural areas? One of the topics that the author is instructing students to study is about the urbanization brought about by Taobao villages. Taobao villages are all located in rural areas, and the products produced by the villagers can be sent to the whole country and even the world. If in a good environment in the countryside, the villagers can do everything here, without having to go to the city, why not do it? However, in a village, it is very difficult for villagers to enjoy better medical services. If the authority wants to retain people in secondary cities, tertiary cities or rural areas, in addition to this limited and fast transportation, various public services must be provided. Why is it that college students would rather drift in the central cities than go to the second and third tier cities?

This involves another problem — the expansion and contraction effect of time and space. China’s urban-rural structure is a kind of network. It used to be based on the village as the junction and spread out. The village went to the town, the town to the county, the county to the prefecture-level city, the prefecture-level city to the provincial capital, and the provincial capital to the central cities, this is called a tandem or a form of tandem. But new road facilities and systems are beginning to break the original link, and any point can be directly connected to other points. This will cause the expansion and contraction effect of this time and space to be very obvious. Why?

Like the example above, the change of traffic routes has weakened the once prosperous cities, and more and more prosperous towns that used to be on the side of the national roads rapidly decline after expressways are built, because the expressways do not pass through them. Through it, it decays rapidly. Examples like this are happening every day, and fast transportation has caused many secondary centres in the past to become suburbs, which can easily become less and less important. This point may become very important for us to think about how to revitalize small and medium-sized cities next. Chongqing and Sichuan are all provinces where migrant workers are exported, and a large number of migrant workers have gone to the coast. Now, if you want to get rich through precise poverty alleviation, you still need to go out to work. One person works and the whole family gets rid of poverty. It is still the same model. Because of the arrival of this kind of rapid transportation, the number of people who go out to work is getting bigger and bigger. In recent years, the population of migrant workers has been a great number every year. In 2020, it was 285 million, in 2019 it was 290 million, and in 2018 it was 288 million (National Bureau of Statistics of China 2020). However, there are fewer and fewer labour-intensive enterprises in the coastal areas, and fewer and fewer jobs can be provided. The contradiction between supply and demand will become larger and larger, and the job market will become tenser and tenser. These are the changes to come brought about by the traffic conditions. There are many people who work in one city and live in another city. How can we revitalize the small and medium-sized cities that are already developed?

In the digital age, the transformation of interpersonal relationships is even more pronounced. We don’t know how human relationships will change in the future. Psychological adaptation and change take a long time. But when we get used to a certain way of life, it will form a kind of inertia. What can be seen now is that the younger generation is unwilling to marry and have children. This is not the same as our Chinese traditions. The family-based society in China in the past may be disintegrating. In the Internet age, such methods of connecting, which used to be based on family units, are no longer suitable for the aborigines of the Internet. Now we are all connected through platforms, using a variety of applications to establish digital connections. While convenient, the problem is also obvious. We personally have no sense of security, and neither do companies. Even if Tencent has reached the top ten in the world, it still has no sense of security, and its rank changes every year. This is the kind of unknown that the digital age brings to people. Because people can’t foresee the future, of course they will have a sense of fear. The Bank of China is going to issue digital currency. The author is a little afraid of this digital currency. If 1 day the computer crashes, will this currency disappear? Of course, digital currency is a global trend, but we are not sure about the digital currency that will appear in the future.

Therefore, the transformation of interpersonal relationships in the digital age has made the interactions between individuals more and more different between families, between generations, between units and between groups. The new connection leads to a change in the actual structure, from a series structure to a parallel structure. In fact, the parallel mode is still an ideal mode, it may become a more diffuse network structure, which is unstable and easy to change. The Blue Book on Cities issued by the Chinese Academy of Social Sciences says that the future cities will develop towards reticular cities (Shan et al. 2020), which is a new trend in China’s urbanization, that is, reticular urban development, which is the formation of inter-reliant networks between regions, furthermore, urban agglomerations. Another key concept of urban agglomeration is that it may be decentralized in the future. We may have good links with each other in the future. Every person and every city will become a core. Of course, this is a topic that is being studied.

The development of a city is also affected by many factors, such as administrative intervention. Cities in China have levels. For example, municipalities directly under the Central Government are generally designated cities under the State Council’s plan, followed by provincial capitals, and then prefecture-level cities and county-level cities. Different levels of cities have different administrative authorities. Cities of the past were tightly integrated with power structures. Decentralization means that the power structure of the city will also change. The political system of the past was built according to the production method of the past, and it was a superstructure constructed corresponding to the productive forces of the past. There is no clear answer to what will happen to our superstructure in the future when the mode of production changes.

In the process of development, China’s cities have established a set of power structures that are not easy to change. The formulation of the power structure is a man-made result and has something to do with human thinking. For example, economists believe that the arrival of globalization will bring about economic integration, but anthropologists believe that the arrival of globalization will strengthen glocalization, localization and nationalization. Take McDonald’s as an example. McDonald’s is an international industry and a global company. Anthropologist James Watson took McDonald’s restaurants in Tokyo, Seoul, Beijing, Shenzhen, and Taipei as research objects. He found that McDonald’s in every city is glocalized (Watson 1988). Therefore, both views are in dispute. Before globalization, not everyone had such a strong sense of national consciousness, nor did everyone have such a strong sense of local consciousness. In the process of globalization, this consciousness is on the contrary stronger. On the surface, many things are becoming more and more consistent, such as mobile phones and the Internet. In depth, on the contrary, when the industry is very consistent and the economy is very consistent, the contradiction between China and the United States is becoming more and more intense, which should have been easier and easier to reach agreement. These are the new problems and topics.

New connection has brought about the globalization of the market. The once local and regional markets in Chinese society can be directly connected to the global market, participating in production and trading. At the same time, they have strong innovation ability because they can accept the news of the world in time. Guzhen Town (meaning ancient town) of Zhongshan City Guangdong Province, for example, is a lighting market, accounting for 70% of the domestic market share and 1/3 of the international market share. This professional town can be connected with the world. Another example is the China Commodity City in Yiwu. If you take a flight from Guangzhou to Yiwu, it will feel like you are on an international flight. Such a small commodity market, through the Internet, it can go to the whole world. The Chinese market benefits from a global connection, and we can see the positive energy.

But there is also a segmented effect. Like the separation brought by the expressway, we can compare each expressway to a river, which will bring the separation effect on both sides. Therefore, the traffic in the city is getting more and more congested, and it is not easy for us to change the traffic congestion. Why? All of us are not walking in the same direction, each of us has different goals. There are constantly all kinds of intersections, all kinds of traffic to cross. And the wider the road is built, the harder it is to cross. This segregation effect is a common phenomenon in daily life.

New topics of anthropology from the perspective of mobility and interlinkage.

In the past, we held view of opposition between urban and rural areas, that is, the city is a big tradition, the countryside is a small tradition, and the small tradition should be subordinate to the big tradition. Digitalization brings about the reconstruction of urban culture or local culture, which everyone can see. The blurring effect of the boundary between urban and rural areas brought about by digitalization will become stronger and stronger. In the Internet age, villages can be linked with the world. Wushi Village, Huzhu Tu Autonomous County, Qinghai is in a very remote place. Young people here host various programs on Kuaishou to promote their embroidery products. These young people can feel the urban lifestyle in the countryside, can compare the spatial and cultural differences between urban and rural areas, and can quickly integrate the urban lifestyle into their own lives, thereby blurring the difference between the two differences.

Another very important point is the splicing of urban and rural cultural integration, which is a topic worthy of study. We used to emphasize the differences between urban and rural cultures. From now on, the influence of the Internet has created a splicing of urban and rural cultures. In the past, anthropologists always wanted to find isolated small groups for research, and Malinowski’s field in the Trobriand Islands was an idealized field landscape. Then, the mobile society has put forward a new proposition for anthropology. Anthropology’s grasp of the cultural dynamics and collective presentation of people’s lives has become a fundamental. Anthropologists must face the problems of transformation in culture in the “liquid society” proposed by Zygmunt Bauman, and understand the self-expression and debugging of culture (Bauman 2018). Therefore, understanding culture can explain the meaning of the whole culture connected with it by using the spliced video as a clue.

The Internet has produced a pair of important concepts: “online” and “offline”. The Internet also gave birth to the guideline of time and space from “offline” to “online”. As you can see, we can directly see people in different places through Kuaishou and Tiktok (Douyin), how they perform, and what way they are, not necessarily popular, but you are willing to watch it because it shows something different from you, if it shows the same thing as you, people will not be interested. In addition, if you look at Kuaishou and Douyin, in the eyes of some artists, they feel that this is not elegant, but their influence is greater than that of ordinary stars, and many professional actors are not convinced. From a cultural perspective, this is a confrontation between popular culture and elite culture, where popular culture has won while elite culture is still at a disadvantage. In the past without the Internet, only stars had the opportunity to show, now everyone has the opportunity to show themselves. In a very remote town in western Hunan, there was an Internet celebrity. His live broadcast appearance fee was very high, and he was paid in seconds. This also provided a new way for social mobility. Therefore, the changes in time and space between online and offline have led to the opportunity for ordinary people who are not professionals or elites to become famous.

We have seen videos about intangible cultural heritage on Kuaishou. There are 40,000 Qin Opera, 790,000 Yangko, 520,000 Dough Kneaders, and 430,000 Henan Opera. On average, every 3 videos have 1 video about intangible cultural heritage (Kuaishou Academy 2020). Kuaishou shows localization and local knowledge, so it is natural to put all kinds of dramas, masks, and performances into it. Cultural heritage is also popular on the platform because of its novelty. On the contrary, no one sees such performances in local society. This is definitely related to the changes in our life rhythm and lifestyle. The more people are interested, the more people will order it and revive what was originally a cultural heritage protection. This is a kind of folk power and a real power. Our current pace of life has become fast, time is fragmented, we are all cut by the Internet, we can only watch short and fast videos, and long videos are not watched.

The video platform represented by Kuaishou has sunk below second-tier cities in a very short period of time. This is the problem of the sinking of Internet platforms. There are various people on Kuaishou platform based on various local cultures. British communication scientist Raymond Williams has a classic theory in his book “Television: Technology and Cultural Forms”, that a material medium already contains a set of social values and concepts. His point of view is enlightening for understanding today’s Internet technology, social needs and existing social conditions create technology, and technology selects cultural forms and self-transforms to express it (Williams 1994). Technology has become a part of today’s social ecology and culture, and the Internet has begun to rebuild a new social and cultural order.

Television has had a great impact on rural life. The author wrote an article about TV and the changes in rural lifestyles (Zhou 1990). But now TV has been replaced by the Internet, and when you go to the countryside, people are watching videos with their mobile phones, no longer watching through TV. Most of the stores in the village, many people gather around to use WIFI. Wherever there’s WIFI, people are gathering around. The shopkeeper must provide free WIFI, otherwise people will be no one sitting around the store and no one doing business with you. It is also a way to attract customers. So now with the emergence of the Internet, all people who want to do Internet business have this idea: how to combine the needs of society with technology. Never before, the technology can be so closely integrated with social life.

Technology is becoming more and more integrated with our life, with our social ecology and culture, and even become a part of it, which was not in the past. The Ministry of Education proposed the idea of new liberal arts, along with the new engineering and the new science, because in the past, engineering students rarely cooperated with liberal arts students, and inventions and creations are likely to have nothing to do with daily life. Now science and technology are more and more closely connected with our culture, our society and our life. That is to say, we now have liberal arts in engineering schools, and engineering in liberal arts schools. This is actually the way of cognition and classification of knowledge by our ancestors needs to change, and led to the emergence of disciplines. Mr. Liu Kuili believes that we used to rely on language or pictures to spread and protect intangible cultural heritage. Now we can pass on, spread and protect intangible cultural heritage through the Internet, and we can do better (Liu 2020). The most important thing about this new technology is that it can break the boundaries of time and space. It’s convenient, and it lets you see anything at anytime, in anywhere.

The digital age has changed the way people connect with society, and has put forward new requirements for understanding society. In the past, we talked about the pattern of differential sequence of Chinese society. When people studied China, they said that China was a society of customs, feelings and faces are the most important concepts. Now since the social foundation has changed, the digital age has created a sense of space compression without distance, privacy is exhibited, privacy is made public, and people have no privacy in front of big data, which has a great impact on everyone. How to define the relationship between Chinese people in the new era has become a new topic for anthropology to think about.

What is the change? The author thinks that it is the sense of unfamiliarity between each other and the sense of distance behind big data. This is a very important feature of our times. On the one hand, we are indeed very cautious with each other, especially with strangers. Another aspect is that the big data behind you know everything about you. If this kind of huge amounts of data is mastered by people with ulterior motives, it will become very dangerous, and the current social risk is greater than in the past. China is in transition, we are transforming from a “regional culture” to a “migrant culture”, from a “single culture” to a “multi-culture”, and from a society of acquaintances to a society of strangers. Generally speaking, in the past, China was a society of acquaintances, whether urban or rural. In the cities after the founding of the People’s Republic of China, the way of the acquaintance society in the countryside was moved to the cities. In China, whether it is the system of neighbourhood committee or work unit, it is a kind of acquaintance society. But now the original acquaintance society has begun to change, because the way we live has changed. Most people no longer live in a work unit, but in the commercial housing community, and the sense of strangeness between residents is getting stronger and stronger. And now all kinds of warnings are telling you not to trust strangers.

In such a digital age, an anonymous age, and a stranger’s age, how to communicate with each other is a new topic and a new task. Our development is so fast that the establishment of the system lags behind. The Internet has brought the convenience of communication, and the number of social networks that we can use technology to socialize has skyrocketed, followed by the generalization of communication caused by overloading.

British Anthropologist Robin Dunbar set a hypothesis. He believes that the number of stable social networks that humans have is 148 people, rounding up, it is about 150 people (Dunbar 2011). But the Internet has brought the convenience of communication. Using network technology, the number of people we socialize on the Internet is still rising, but it also directly leads to overloading. Information overload is the problem we all face now, because information overload will affect the trust mechanism of yours.

Now there’s a buzzword in today’s China: involution. In the Internet age, people seem to be less and less afraid to make new friends, so is it that we are also involution in social interaction? This is a big topic that can be studied, that is, why our communication is involution, and why are we unwilling to do so now to make new friends, and just swirl around in a circle of old acquaintances.

## Conclusion: how does anthropology respond to change?

The covid-19 epidemic has highlighted the effectiveness of grid management, and now China is implementing community management in many places. However, this also brings a great management paradox. If everyone is in the grid, then the authority knows the mobility of everyone very well. But once people are moving, connecting, with the existing technology, the mobility of people has been strengthened like never before. How can the authority manage? And how can the society achieve better governance? In real life, many factors are beyond our expectations.

The convenience and acceleration of transportation has made the mobility of people and materials unprecedented in both speed and scale, bringing not only changes in lifestyles, but also demographic changes and cultural transformations; this cultural transformation will lead to traditional anthropology changes in research objects, the basic theories, methods and concepts of the anthropology are all facing this adjustment and new challenges (Zhou 2017).

The Internet is a new scope, and it is a new attempt for anthropology to enter the Internet research. However, the exploration of related research is inseparable from the innovative spirit of all researchers and the inclusive and open academic attitude. As far as basic knowledge and practical traditions are concerned, the research approach of Internet anthropology has an inheritance relationship with traditional anthropology research. At the same time, Internet anthropology research also has obvious intersections with different branches of today’s anthropology, such as urban anthropology, development anthropology, medical anthropology, media anthropology, etc. The research in these sub-fields has begun to take the Internet as an important variable, which requires the support and cooperation of Internet anthropology. The development of Internet anthropology provides not only a more forward-looking academic perspective, but also a platform that can integrate multiple resources, including academia, industry, and government, and facilitate communication and dialogue within and among disciplines, as well as between academia, business and government. In the foreseeable future, Internet anthropology will make outstanding contributions.

## Data Availability

Not applicable.
